# The Odon Device™ for assisted vaginal birth: a feasibility study to investigate safety and efficacy—The ASSIST II study

**DOI:** 10.1186/s40814-021-00814-2

**Published:** 2021-03-19

**Authors:** Emily J. Hotton, Mary Alvarez, Erik Lenguerrand, Julia Wade, Natalie S. Blencowe, Tim J. Draycott, Joanna F. Crofts, The ASSIST II Study Group, Mary Alvarez, Sabaratnam Arulkumaran, Nichola Bale, Natalie S. Blencowe, Joanna F. Crofts, Timothy J. Draycott, Mohamed Elhodaiby, Lily Exell, Islam Gamaleldin, Simon Grant, Sally Hall, Cameron Hinton, Emily J. Hotton, Hajeb Kamali, Erik Lenguerrand, Abi Loose, Helen Lewis-White, Naomi Mallinson, Glen Mola, Alison Pike, Rachel Powell, Iona Smith, Claire Rose, Julia Wade, Paul White, Kathryn Walpole, Cathy Winter

**Affiliations:** 1 Translational Health Sciences, Bristol Medical School, University of Bristol, Bristol, BS10 5NB UK; 2 Women & Children’s Directorate, North Bristol NHS Trust, Bristol, UK; 3 Population Health Sciences, Bristol Medical School, University of Bristol, Bristol, UK; 4 Centre for Surgical Research - Population Health Sciences, University of Bristol, Bristol, UK; 5 NIHR Bristol Biomedical Research Centre, University Hospitals Bristol NHS Foundation Trust, Bristol, UK

**Keywords:** Assisted vaginal birth, Obstetrical vacuum extraction, Obstetrical extraction, Odon Device, Obstetrical forceps, Feasibility studies, Device safety, Medical devices

## Abstract

**Background:**

The Odon Device™ is a new device for assisted vaginal birth that employs an air cuff around the fetal head for traction. Assisted vaginal birth (AVB) is a vital health intervention that can result in better outcomes for mothers and their babies when complications arise in the second stage of labour. Unfortunately, instruments for AVB (forceps and ventouse) are often not used in settings where there is most clinical need often due to lack of training and resources, resulting in maternal and neonatal morbidity and mortality which could have been prevented.

This is often due to a lack of trained operators as well as difficulties in the sterilisation and maintenance of AVB devices. This novel, single use device has the potential to mitigate these difficulties as it is single use and is potentially simpler to use than forceps and ventouse.

All the studies of the Odon Device to date (pre-clinical, preliminary developmental and clinical) suggest that the Odon Device does not present a higher risk to mothers or babies compared to current standard care, and recruitment to intrapartum research exploring the device is feasible and acceptable to women. The first study in which the Odon Device was used in clinically indicated conditions (the ASSIST Study) reported a lower efficacy than those reported with established devices. The reasons need to be explored, specifically focussing on learning curve, the technique of the doctors using this new device and potential modifications to device design. A follow-on clinical study to further investigate the efficacy and safety of the Odon Device in its indicated use, the ASSIST II Study, is therefore being undertaken.

**Methods:**

The primary feasibility outcome is study feasibility (recruitment and retention rates) whilst the primary clinical outcome successful vaginal birth completed with the Odon Device. Key secondary feasibility outcomes include participant withdrawal, compliance in data collection and acceptability of the device to women and operators. Secondary clinical outcomes include maternal, neonatal and device outcomes. Safety data will be reviewed following every birth exploring maternal, neonatal and device risks. Using A’Hern approach for sample size calculation, we aim to recruit 104 women requiring an assisted vaginal birth for a recognised clinical indication. Assuming an AVB success rate of 65% or more, a one-sided alpha risk of 5% and power of 90%.

**Discussion:**

The data from the ASSIST II Study will provide the information required regarding acceptability, recruitment, outcome data collection, device design, technique of device use and operator learning curve in order to design a future randomised controlled trial of the Odon Device versus current modes of assisted vaginal birth.

**Trial registration:**

ISRCTN registration: 38829082 (prospectively registered July 26, 2019)

**Supplementary Information:**

The online version contains supplementary material available at 10.1186/s40814-021-00814-2.

## Background

No new methods of assisting vaginal birth have been introduced into clinical practice since the development of the ventouse in the 1950s, despite the huge advances in medical care over the past 70 years. Complications of the second stage of labour remain a major cause of maternal and neonatal morbidity and mortality. Assisted vaginal birth (AVB) reduces adverse outcomes for women and their babies relative to a caesarean section performed in the second stage of labour [1]. There is significant variation in the rates of AVB worldwide, with many low- and middle-income countries reporting that AVB is not utilised at all [2]. AVB is currently performed regularly (10 to 15% of births) in the UK, Spain, France, Ireland, Portugal, Canada and Australia, occasionally in the Netherlands, Sweden and Algeria (5 to 10% of births), and rarely (less than 5% of births) in the USA, India and most low- and middle-income countries [3–6].

Currently, a vaginal birth may be expedited through the use of obstetric forceps or ventouse. Forceps are more likely to achieve an AVB when compared to a ventouse; however, they are associated with increased maternal perineal and vaginal trauma. The ventouse is more likely to fail than forceps and is associated with an increased risk of neonatal cephalohaematoma and retinal haemorrhage [7].

**Fig. 1:**
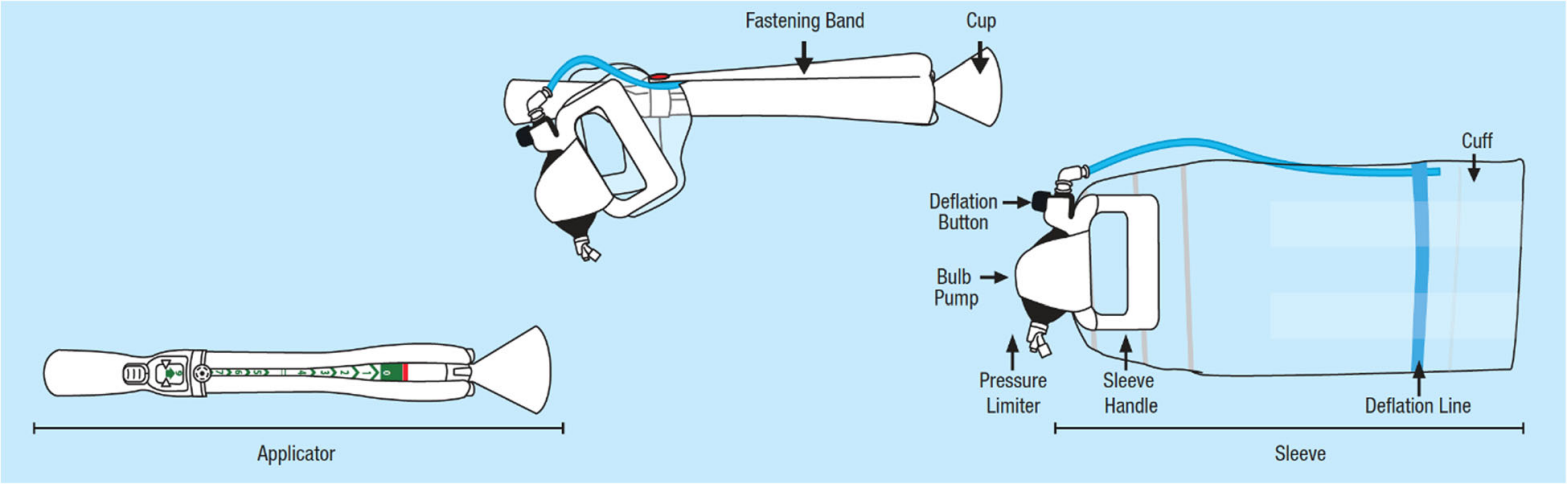
The Odon Device component parts

The Odon Device is a new device for AVB, comprising two main components: a polyethylene sleeve and a plastic applicator (Fig. 1). The applicator consists of a handle with four pronged flexible spatulas that slide around the fetal head to position the sleeve. The applicator has a progress indicator allowing the operator to check when the correct depth of insertion has been reached. The sleeve contains a circumferential air chamber that is inflated around the fetal head. The use of an air chamber to act as the traction point on the fetal head is hypothecated to reduce adverse events associated with the greater pressures applied to the fetal head during the use of forceps. The lack of negative pressure on the fetal head, the mechanism of action of the ventouse, is designed to eliminate vacuum-associated haematoma and haemorrhage. These contentions have been supported in pre-clinical simulation studies [8–10].

The first in human pilot study of four earlier versions of the Odon Device in 49 healthy volunteers in Argentina and South Africa [11]. Further extensive simulation and human factors studies were performed to investigate safety and usability [12].

The ASSIST Study (conducted between October 2018 and April 2019) was the first study in which the Odon Device was used to assist vaginal birth for clinically indicted conditions (e.g. in cases of prolonged second stage of labour or presumed fetal compromise) [13, 14].

All the studies of the Odon Device to date (pre-clinical, preliminary developmental and clinical) suggest that the Odon Device does not present a higher risk to mothers or babies compared to current standard care (forceps and ventouse). However, the number of births assisted to date with the device is small and more experience of using the device is required to generate an evidence based safety profile. Furthermore, the reasons for the lower than expected success rate of the device needs to be explored, specifically focussing on obstetrician learning curves and the potential need for modifications to device design. A follow-on feasibility study to further investigate the efficacy and safety of the Odon Device in its indicated use, the ASSIST II Study has therefore been designed.

## Methods/design

The main aim of the study is to assess the feasibility of conducting a definitive trial in terms of recruitment and acceptability of the intervention, follow-up and data collection methods. In addition, the study aims to explore successful vaginal birth completed with the Odon Device.

### Objectives

(i) To estimate feasible eligibility, recruitment as well as refusal and follow-up rates
(ii) To investigate the feasibility of collecting data as well as exploring the safety and efficacy of forceps and ventouse from women who did not receive the Odon Device
(iii) To investigate the safety, efficacy and acceptability of a new device for the Odon Device
(iv) To define the clinical circumstances in which the device is most effective.
(v) To study the learning curve of the operator and to investigate the most effective user technique.
(vi) To study the device design and functionality.

### Study design

The ASSIST II Study (Assisted Vaginal Birth Study) is a non-randomised feasibility study of women who require an AVB for a recognised clinical indication. The study also includes a nested cohort study of women and babies who had a ‘missed’ Odon Device assisted birth. These cases comprise women who have consented to participate in the ASSIST II Study and require an AVB but are unable to have a Odon Device assisted birth due to operator availability. The inclusion of the nested cohort will explore the feasibility of collecting information from a ‘comparator group’. The information gained from this study will be used to plan a large randomised controlled trial that will directly compare the Odon Device with the ventouse.

The key changes to the ASSIST II Study protocol as compared to the ASSIST Study protocol are as follows:

- Refined participant inclusion criteria to exclude very complex births when the vertex is at the level of the ischial spines.
- Inclusion of a nested cohort of ‘missed’ births.
- Larger sample size to support investigation into efficacy (104 births compared to 40).
- Manufacturer will ensure the device is manufactured to the required specification to avoid malfunction of the bulb pump experienced in the ASSIST Study.
- A simplified clinical primary outcome.
- Streamline changes to the data collection forms.

**Fig. 2:**
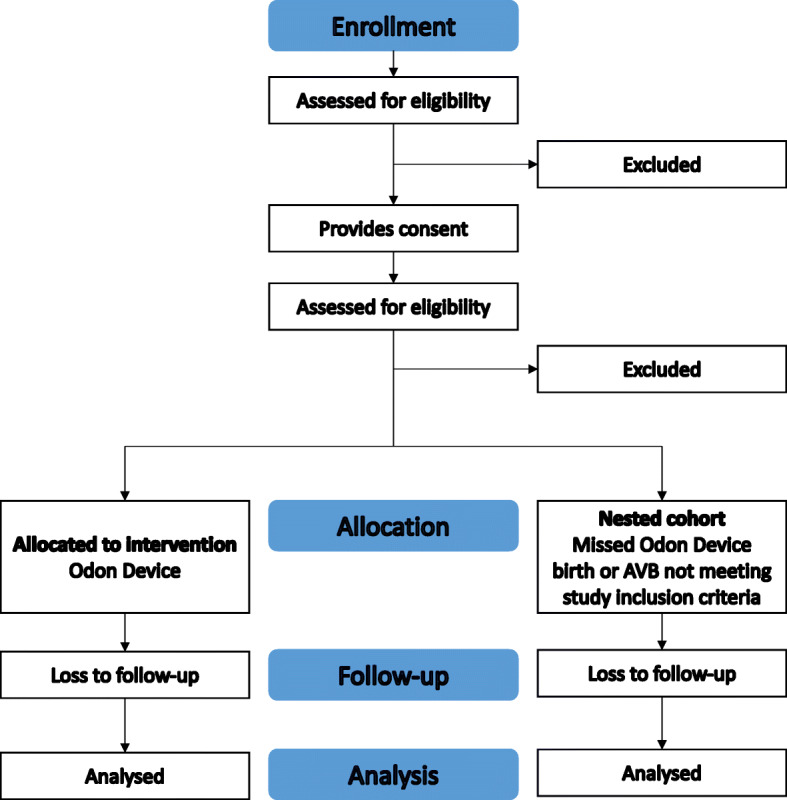
CONSORT diagram of the ASSIST II Study

A CONSORT diagram of the ASSIST II Study is shown in Fig. 2.

### Population/sample

Participants will be pregnant women aiming for a vaginal birth who plan to give birth at North Bristol NHS Trust (NBT), Bristol, UK. Recruitment is projected to continue for 11 months (due to the AVB rate within the department) after which time it is estimated that 104 sets of clinical primary outcome data will have been recorded.

**Fig. 3:**
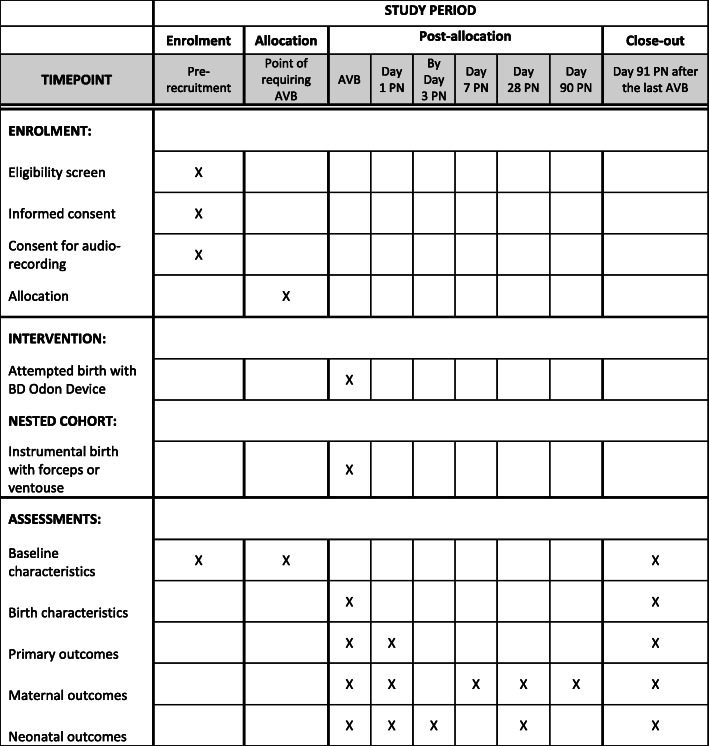
SPIRIT figure: schedule of enrolment, interventions and assessments

Prospective participants will receive information on the study in early pregnancy (12 to 28 weeks) via the NBT Maternity ‘App’ (this ‘App’ is provided to all pregnant women at NBT and provides information on all aspects of their maternity care) and paper information leaflets, given to women at any hospital admission. Members of the study team will then approach women after 28 completed weeks of pregnancy during antenatal appointments or antenatal admissions, to discuss the study and offer women the opportunity to watch a video explaining the study. Women who are willing to take part (should they require an AVB) will then be invited to provide informed written consent. Figure 3 demonstrates the SPIRIT figure outlining the schedule of enrolment, interventions and assessments. SPIRIT checklist can be found in Additional file 1.

When a woman who has previously consented to participate in the study arrives on the labour ward, her eligibility to participate in the study will be rechecked and verbal re-confirmation of her consent to take part in the study will be sought by a midwife and obstetrician.

### Inclusion and exclusion criteria

Women will be able to participate in the ASSIST II Study if all of the following apply at initial consent: ≥18 years of age; singleton pregnancy of at least 36 weeks’ gestation; negative antenatal screen for HIV and Hepatitis B; in active labour and requiring an assisted vaginal birth for a clinical indication (as per the Royal College of Obstetricians & Gynaecologists (RCOG) Greentop Guideline 26) [7]: the vertex is 1 cm or more below the ischial spines; the woman has effective analgesia in place during the use of the instrument (i.e. epidural, spinal or pudendal block, or perineal infiltration with local anesthetic), and there is no obstetric indication for an alternative method of AVB).

Women will not be able to take part in the ASSIST II Study if there is the fetal vertex at or above the ischial spines, a diagnosis of a fetal skull abnormality (i.e. macrocephaly) or osteogenesis imperfecta, suspicion of a fetal bleeding disorder (von Willebrand’s disease, AITP, haemophilia), an intrauterine fetal death in the current pregnancy, the woman is sensitive to latex, the woman is currently serving a prison sentence, and there is a fetal bradycardia which is on-going and has not recovered.

### Intervention

If the woman agrees to an AVB, an accoucheur who has had specific training in using the Odon Device will assist the birth with the Odon Device. Should the birth not be achieved with the Odon Device, the obstetrician will use their clinical judgement on an individual case basis to complete the birth using ventouse, forceps or caesarean section as appropriate. Primary, secondary and safety data will be gathered regarding the assisted birth and use of the device with additional qualitative investigation of recruitment processes to the study.

### Nested cohort

If an obstetrician who has had specific training in using the Odon Device is not available to attend the birth of an eligible and consented woman, the woman and her baby will be included in the nested cohort group. The birth will be assisted by the on-call team as per routine care in the maternity unit. All birth and outcome data will be gathered regarding the assisted birth and use of forceps and/or ventouse.

### Outcomes

#### Feasibility primary outcome

Study feasibility through descriptions of recruitment and retention rates of women who may require an AVB at the end of their labour.

### Additional feasibility outcomes

As well as recruitment rates, the following feasibility outcomes will be explored in detail:

- Follow-up rates and reasons for declining or withdrawal (medical, personal, logistic, others).
- Suitability of outcome measures and overall data collection processes, including complete list of data collection and recruitment rate to the study.
- Ability to collate data from a ‘nested’ cohort will help demonstrate whether collecting data on a control group in a RCT is feasible.

Patient acceptability will be assessed through the patient perception score of birth experience days 1, 7 and 28 postnatal (score out of 15 where 3 is the lowest score possible) and through qualitative assessment.

Practitioner acceptability will be assessed through perceived outcomes following each attempted AVB. The following will be collected on a 5-point Likert scale.

- Instrument of choice if Odon Device was not used (rotational forceps/non-rotational forceps/KIWI ventouse/silastic ventouse).
- Perceived overall ease of use of device (for all AVBs).
- Ease of device set-up (for all AVBs).
- Ease of device application to the baby’s head (for all AVBs).
- Ease of withdrawal of the applicator after application (for Odon births only).
- Comfort with the level of force required to assist the birth of the baby (for all AVBs).
- Ease of deflation of the cuff prior to crowning (for Odon births only).
- Ease of removal of the forceps or ventouse.

In order to further understand the technique for device use, any birth with the Odon Device, the practitioner (and research midwife if present) will document the angle of device application using a grid stating whether the handle of the device is pointing: up, down or horizontally.

### *Clinical primary outcome:* proportion of births successfully assisted with the Odon Device

A birth will be defined as ‘successful’ if all of the following criteria are met:

1. The birth of the baby is expedited with the Odon Device.
2. There are no serious maternal adverse reactions related to the use of the device during birth.
3. There are no serious neonatal adverse reactions related to the use of the device during birth (Table 1).

**Table 1:** Primary outcome for the ASSIST II Study

Criteria	Source	Collected by
The birth of the baby is expedited with the Odon Device	AVB pro forma Medical notes	Research team member including device operator
There are no serious maternal adverse events related to the use of the device during birth	AVB pro forma Medical notes	Research team member including device operator
There are no serious neonatal adverse events related to the use of the device during birth	AVB pro forma Medical notes	Research team member including device operator

### Secondary clinical outcomes

Secondary outcome measures are categorised below and include measures needed to ascertain the feasibility of a future RCT:

### Birth

- Time from ‘decision to perform assisted birth’ to ‘birth’ (minutes).
- Time from ‘device application” to ‘birth’ (minutes).
- Time between ‘decision to perform assisted birth’ to ‘time of Odon application’.
- Location of birth.
- Mode of birth following failed use of Odon device.

### Device

- Failure of a component of the Odon Device.
- Number of applications of device.
- Number of pulls of the device.
- Any trauma caused by first device application.
- Perceived reason for device failure from operator (maternal/clinical/device/other).

### Maternal

- Weighed blood loss.
- Perineal and anal sphincter injury (1st/2nd/3rd/4th degree tears and episiotomy).
- Ischio-rectal fossa defect.
- Cervical tear (present not requiring suturing).
- Cervical tear (present requiring suturing).
- Labial tear requiring suturing.
- Lower segment caesarean section performed (Yes/No).
- Use of analgesia days 1, 7 and 28 postnatal.
- Details of analgesia used at days 1, 7 and 28 postnatal.
- Maternal death.
- Length of hospital stay following birth.

### Neonatal

- Umbilical artery pH and base excess.
- Umbilical vein pH and base excess.
- Shoulder dystocia.
- Apgar scores at 1, 5 and 10 min post birth.
- Neonatal Infant Pain Scores at 2 and 6 h post birth.
- Neonatal feed by 10 h post birth.
- Method of feeding days 1, 7, 28 and 90 post birth.
- Admission to Neonatal Intensive Care Unit (NICU).
- Time spent in NICU (hours).
- Neonatal soft tissue trauma (bruise/scalp/facial injury/cephalohaematoma).
- Neonatal vascular injury (subaponeurotic haemorrhage).
- Neonatal body maps (head and neck).
- Neonatal skeletal injury (bone fracture).
- Neonatal intra-cranial injury (cerebral contusion).
- Neonatal neurological injury still present at day 28 post birth.
- Neonatal seizure by day 28 post birth.
- Phototherapy for jaundice contributed to by bruising by day 28 post birth.
- Anaemia requiring transfusion by day 28 post birth.
- Neonatal encephalopathy requiring therapeutic hypothermia within 28 days post birth.
- Organ failure within 28 days post birth.
- Other neonatal injury.
- Neonatal death within 28 days post birth.

### Patient-reported outcomes

- Maternal health-related quality of life data (EQ-5D-5L) will be collected during the antenatal period and at day 1 and day 28 postnatal.
- Maternal perception of pain (11-point Likert scale) at days 1, 7 and 28 postnatal.
- Maternal continence at day 90 postnatal.

### Health utilisation form outcomes

Data regarding visits to a health care professional in either the hospital or community setting within the first 28 days postnatal will be collected.

### Safety of intervention

A comprehensive assessment of the safety of the Odon Device will be undertaken following every attempted birth within the ASSIST II Study. Outcome measures and data collected will ensure capture of any potential adverse events associated with the Odon Device at time of birth by the operator and/or a member of the research team. All follow-ups will be performed by the research team. In the immediate postpartum period a member of the research team will follow-up the participant on a daily basis until discharge, collecting the day one data. To ensure that any serious adverse events that occur in the postnatal period are captured, an Adverse Event Reporting System will be initiated, for ward staff to highlight any adverse events that occur after birth. In addition, device failure (or failure of any component) will be reported as an individual outcome measure. Follow-ups at days 7, 28 and 90 will be conducted by a member of the research team telephoning the woman using their contact details provided to the study team. The Trial Management Group (TMG) and Sponsor will regularly review data from all births to ensure early identification of any trends of adverse events. All adverse events will be classified and reported according to the schedule of the Medicines and Healthcare Regulation Agency (MHRA) and the Research Ethics Committee.

### Patient and public involvement

Women and their partners have been involved in the development of the feasibility ASSIST II Study prior to the submission for research approvals—patient and public involvement (PPI) has reviewed the proposed study design, as well as woman-facing documentation (leaflets, videos and consent forms) and has supported both the general and specific aims of the study. A lay patient representative will be a member of the Trial Steering Committee (TSC) for the ASSIST II Study.

### Analysis

Investigators had established a priori threshold for specific feasibility and acceptability criteria. These were the following: (i) the proportion of eligible women agreeing to participate in the study would be 54% or greater (the threshold for the first clinical trial of the Odon Device [11]), (ii) retention would be 90% of greater, (iii) at least 95% of women had a positive birth satisfaction score at day one postnatal and (iv) at least 95% of practitioners had a positive opinion on the use of the Odon Device. The feasibility outcomes will be reported descriptively and narratively. Reasons for ineligibility, refusal, lost-to-follow-up or missing data will be categorised and described as frequencies. Women and their babies will be followed up at 1 day, 7 days, 28 days and 90 days following the birth. A woman and her baby will be deemed to complete their participation in the study at 90 days after the birth.

Clinical outcomes will be reported as frequency and proportion, mean (standard deviation) or median (interquartile range) depending on their nature and distribution. The overall rate of successful AVB birth will be reported and the frequency of unsuccessful births will be reported by the three criteria defined for success. The overall number of safety events will be reported, as well as the number of events, by main reasons for adverse events. Events related to the device failure and/or mis-use of the device will also be described.

### Integrated qualitative research

In ASSIST II Study the integrated qualitative study (IQS) will focus exclusively on optimum processes for providing information about and obtaining informed consent for research studies that involve interventions that may be initiated in the intrapartum period.

The aims of the IQS are to evaluate the model of information provision and receiving informed consent developed for the ASSIST II (comprising the ASSIST II Study information video embedded within informed consent discussions with research midwives, developed based on findings of a study evaluating information provision processes within the ASSIST Study).

Data will include audio-recordings of recruitment discussions between research midwives and women eligible to participate in the study and in-depth interviews with both women who have been invited to participate in the study and the research midwives involved in inviting them to participate. Written consent from all participants will be obtained prior to any qualitative research and verbal re-confirmation of consent will be obtained prior to each audio recording and interview. Findings will inform information provision during recruitment to any future study involving the Odon Device.

### Sample size

Although a formal sample size is not required, the A’Hern approach for sample size calculation was performed in PASS (Power and Sample Size) software to help judgement regarding an appropriate figure. Assuming that the success rate (P) of a poor AVB is 50%, and the success rate of a good AVB would be 65% or more, and one-sided alpha risk of 5% and power of 90%, a study with 104 participants will be required to decide whether the success rate of the Odon Device is less than or equal to 50%, or greater than or equal to 65%. The success rate of 65% was identified from Attilakos et al., an AVB study conducted in the same unit as the ASSIST II Study which reported a success rate for a single-use, hand-held ventouse device of 66% [15].

An interim safety analysis will be conducted to monitor and check the maternal and neontatal safety data only; no assessment of the Odon Device success will be conducted at this interim point. The whole sample size (*n*=104) will be required to pursue the understanding of the device used in the clinical context of the study. This safety interim analysis will be performed around the 42nd attempted birth using the Odon Device.

### Trial oversight

The TMG consisting of all investigators, co-investigators and Sponsor will be responsible for the day-to-day running of the study. The study will be overseen by two committees, the Independent Data Monitoring Committee (IDMC) and the Trial Steering Committee (TSC). The IDMC will sit after the 42nd birth has been attempted using the Odon Device. The IDMC will have no direct involvement in the running of the trial. The IDMC will generate a report on the performance of the device and safety of participants. The TSC will consist of an independent clinical expert, statistician and a lay representative, together with the investigators and Sponsor representative. The TSC will review all reports produced by the IDMC and make a recommendation to the Sponsor following every review to continue, modify or halt the study. The TSC will provide oversight of the progress of the study and ensure the study is conducted according to the principles of Good Clinical Practice (GCP). Auditing will take place when requested by the Sponsor.

### Dissemination

Study results will be published within one year of completion of data collection in an appropriate peer-reviewed open-access journal. The results will be presented at local, national and international meetings. Summaries will also be distributed using existing parent networks. A summary of results will also be sent to all women who participated in the study (including nested cohort participants), unless they express their wish not to receive such information. Results will be communicated to a lay audience by social media activities of North Bristol NHS Trust, University of Bristol and the research team.

## Discussion

An appropriately conducted AVB, performed when clinically indicated, is associated with improved maternal and neonatal outcomes when compared to caesarean section in the second stage of labour or no action [1]. The ASSIST Study has demonstrated that it is feasible to recruit women to an intrapartum research study exploring novel device for AVB, with women, obstetricians and midwives finding the device acceptable. There has been no evidence of an increased risk to mothers or babies when comparing the Odon Device to forceps or ventouse [13].

The success rate of the Odon Device was lower than the reported success rates of both forceps and vacuum and that of the initial pilot study [7, 11, 15]. However, the ASSIST Study was the first time that the Odon Device had been used in clinically indicated conditions. Aspects that need to be further explored include the clinical circumstances in which the device is used, the technique of application and traction, the operators prior experience of the device (learning curve) and the design and functionality of the device itself. We believe that it is therefore reasonable to proceed to a further feasibility study of the Odon Device, and, if positive, a randomised controlled trial.

If found safe by the TSC, IDMC and Sponsor, findings from the ASSIST, ASSIST II Studies and parallel feasibility study in France [16] will inform the design of a randomised controlled trial that may provide evidence that supports the introduction of the device into clinical practice as a new device for AVB.

### Trial status

The ASSIST II Study commenced on the 9th of August 2019, using version 0.2 of the protocol. The study will cease recruitment after 104 complete sets of primary outcome data are received. This is projected to be in June 2020.

## Supplementary Information

**Additional file 1.** SPIRIT 2013 checklist.

**Additional file 2.** Ethics Committee Approval for the ASSIST II Study Format.

**Additional file 3.** Confirmation of funding for the ASSIST II Study.

## Data Availability

All anonymised data and results of analyses will be available on a publicly accessible database hosted by the University of Bristol. The authors have used the Spirit Checklist 2013 to ensure the relevant details are included in the Clinical Trial Protocol (Additional file 1).

## Abbreviations

AVB: Assisted vaginal birth
BD: Becton, Dickinson and Company
GCP: Good clinical practice
IDMC: Independent Data Monitoring Committee
IQS: Integrated qualitative study
LMIC: Low- and middle-income countries
MHRA: Medicines and Healthcare products Regulatory Agency
NBT: North Bristol NHS Trust
NIPS: Neonatal Infant Pain Score
PPI: Patient and public involvement
RCOG: Royal College of Obstetrics and Gynaecology
RCT: Randomised controlled trial
TMG: Trial Management Group
TSC: Trial Steering Committee
UoB: University of Bristol
