# Supplementary material for: The Odon Device™ for assisted vaginal birth: a feasibility study to investigate safety and efficacy—The ASSIST II study
Source: Pilot Feasibility Stud. 2021 Mar 19;7:72. doi: 10.1186/s40814-021-00814-2 (PMC7977305; doi:10.1186/s40814-021-00814-2)
Supplement: Supplementary file 2 — Additional file 2. Ethics Committee Approval for the ASSIST II Study Format. [file 40814_2021_814_MOESM2_ESM.pdf]

Health Research Authority

South Central - Berkshire Research Ethics Committee
Bristol REC Centre
Whitefriars
Level 3, Block B
Lewins Mead
Bristol
BS1 2NT

Telephone: 0207 104 8241

Please note: This is the favourable opinion of the REC only and does not allow you to start your study at NHS sites in England until you receive HRA Approval

29 May 2019

Professor Timothy Draycott
Professor of Obstetrics
North Bristol NHS Trust
The Chilterns
Southmead Hospital
Bristol
BS10 5NB

Dear Professor Draycott

**Study title:**	**The BD Odon Device for assisted vaginal birth: a feasibility study to investigate safety and efficacy**
**REC reference:**	**19/SC/0226**
**Protocol number:**	**0.1**
**IRAS project ID:**	**264660**

The Research Ethics Committee reviewed the above application at the meeting held on 21 May 2019. Thank you to the Consultant Obstetrician, Dr Jo Crofts, Consultant Neonatologist, Dr Alison Pike, and Senior Research Midwife, Ms Mary Alvarez for attending to discuss the application.

We plan to publish your research summary wording for the above study on the HRA website, together with your contact details. Publication will be no earlier than three months from the date of this favourable opinion letter. The expectation is that this information will be published for all studies that receive an ethical opinion but should you wish to provide a substitute contact point, wish to make a request to defer, or require further information, please contact hra.studyregistration@nhs.net outlining the reasons for your request. Under very limited circumstances (e.g. for student research which has received an unfavourable opinion), it may be possible to grant an exemption to the publication of the study.

Ethical Opinion

The members of the Committee present gave a favourable ethical opinion of the above research on the basis described in the application form, protocol and supporting documentation, subject to the conditions specified below.

Conditions of the Favourable Opinion

The REC favourable opinion is subject to the following conditions being met prior to the start of the study.

Number	Condition
1	Add the sentence "Should you consent to participate in the study, but are not selected to receive the device, then we may still call upon you and/or use your data" to the Participant Information Sheet, as currently the nested group concept is not clear.

You should notify the REC once all conditions have been met (except for site approvals from host organisations) and provide copies of any revised documentation with updated version numbers. Revised documents should be submitted to the REC electronically from IRAS. The REC will acknowledge receipt and provide a final list of the approved documentation for the study, which you can make available to host organisations to facilitate their permission for the study. Failure to provide the final versions to the REC may cause delay in obtaining permissions.

Management permission must be obtained from each host organisation prior to the start of the study at the site concerned.

Management permission should be sought from all NHS organisations involved in the study in accordance with NHS research governance arrangements. Each NHS organisation must confirm through the signing of agreements and/or other documents that it has given permission for the research to proceed (except where explicitly specified otherwise).

Guidance on applying for HRA and HCRW Approval (England and Wales)/ NHS permission for research is available in the Integrated Research Application System, at www.hra.nhs.uk or at http://www.rdforum.nhs.uk.

Where a NHS organisation's role in the study is limited to identifying and referring potential participants to research sites ("participant identification centre"), guidance should be sought from the R&D office on the information it requires to give permission for this activity.

For non-NHS sites, site management permission should be obtained in accordance with the procedures of the relevant host organisation.

Sponsors are not required to notify the Committee of management permissions from host organisations.

Registration of Clinical Trials

All clinical trials (defined as the first four categories on the IRAS filter page) must be registered on a publically accessible database. This should be before the first participant is recruited but no later than 6 weeks after recruitment of the first participant.

There is no requirement to separately notify the REC but you should do so at the earliest opportunity e.g. when submitting an amendment. We will audit the registration details as part of the annual progress reporting process.

To ensure transparency in research, we strongly recommend that all research is registered but for non-clinical trials this is not currently mandatory.

If a sponsor wishes to request a deferral for study registration within the required timeframe, they should contact hra.studyregistration@nhs.net. The expectation is that all clinical trials will be registered, however, in exceptional circumstances non registration may be permissible with prior agreement from the HRA. Guidance on where to register is provided on the HRA website.

It is the responsibility of the sponsor to ensure that all the conditions are complied with before the start of the study or its initiation at a particular site (as applicable).

Ethical review of research sites

NHS Sites

The favourable opinion applies to all NHS sites taking part in the study taking part in the study, subject to management permission being obtained from the NHS/HSC R&D office prior to the start of the study (see "Conditions of the favourable opinion" below).

Non NHS sites

The Committee has not yet completed any site-specific assessment(s) (SSA) for the non-NHS research site(s) taking part in this study. The favourable opinion does not therefore apply to any non-NHS site at present. I will write to you again as soon as an SSA application(s) has been reviewed. In the meantime no study procedures should be initiated at non-NHS sites.

Approved Documents

The documents reviewed and approved at the meeting were:

Document	*Version*	*Date*
Copies of advertisement materials for research participants [Poster]	0.1	21 March 2019
GP/consultant information sheets or letters [GP letter]	0.1	22 March 2019
Instructions for use of medical device [IFU]	01(A)	29 March 2019
Interview schedules or topic guides for participants [Participant]	1	20 March 2019
Interview schedules or topic guides for participants [Midwife and doctor]	1	20 March 2019
IRAS Application Form [IRAS_Form_08042019]		08 April 2019
IRAS Application Form XML file [IRAS_Form_08042019]		08 April 2019
IRAS Checklist XML [Checklist_09052019]		09 May 2019
Other [Follow-up]	0.1	22 March 2019
Other [Consenting video]	0.1	22 March 2019
Other [Contact form]	0.1	20 March 2019
Other [Sent to patients if do not answer the phone]	0.1	22 March 2019
Other [Withdrawal]	0.1	22 March 2019
Other [Cover letter]	1	05 April 2019

Other [Initial maternal data collection form]	0.1	22 March 2019
Participant consent form [Midwife and doctor]	0.1	22 March 2019
Participant consent form [Participant]	0.1	22 March 2019
Participant consent form [Participant qualitative]	0.1	22 March 2019
Participant information sheet (PIS) [PIL]	0.1	20 March 2019
Participant information sheet (PIS) [Midwife and doctor]	0.1	22 March 2019
Research protocol or project proposal [Protocol]	0.1	04 April 2019
Summary CV for Chief Investigator (CI) [CV]	2	04 April 2019
Validated questionnaire [EQ-5D-5L]		

Membership of the Committee

The members of the Ethics Committee who were present at the meeting are listed on the attached sheet.

The Committee is constituted in accordance with the Governance Arrangements for Research Ethics Committees and complies fully with the Standard Operating Procedures for Research Ethics Committees in the UK.

After Ethical Review

Reporting requirements

The attached document "After ethical review – guidance for researchers" gives detailed guidance on reporting requirements for studies with a favourable opinion, including:

- Notifying substantial amendments
- Adding new sites and investigators
- Notification of serious breaches of the protocol
- Progress and safety reports
- Notifying the end of the study

The HRA website also provides guidance on these topics, which is updated in the light of changes in reporting requirements or procedures.

User Feedback

The Health Research Authority is continually striving to provide a high quality service to all applicants and sponsors. You are invited to give your view of the service you have received and the application procedure. If you wish to make your views known please use the feedback form available on the HRA website: http://www.hra.nhs.uk/about-the-hra/governance/quality-assurance/

HRA Learning

We are pleased to welcome researchers and research staff to our HRA Learning Events and online learning opportunities– see details at: https://www.hra.nhs.uk/planning-and-improving-research/learning/

**19/SC/0226**	**Please quote this number on all correspondence**

With the Committee's best wishes for the success of this project.

Yours sincerely

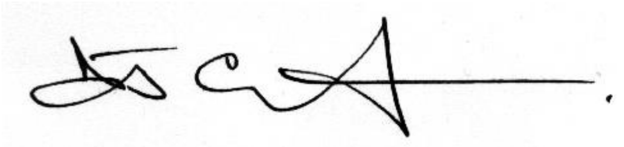

Mr David Carpenter
Chair

E-mail: nrescommittee.southcentral-berkshire@nhs.net

Enclosures: *List of names and professions of members who were present at the meeting and those who submitted written comments*

Copy to: *Dr Emily Hotton*

South Central - Berkshire Research Ethics Committee

Attendance at Committee meeting on 21 May 2019

Committee Members:

Name	*Profession*	*Present*	*Notes*
Mr David Carpenter (Chair)	Retired Social Scientist	Yes	
Dr Mike Emanuel	Pharmaceutical Consultant	No	
Mr Martin Hopkinson	Director of risk management services	Yes	
Mrs Liz Hunter	Retired Midwife and Clinical Governance Manager	No	
Dr Vandana Luthra	R&D Research Co-ordinator	Yes	
Mr Daniel Charles Mace	Retired Corporate Lawyer	Yes	
Mr Richard Merewood	Director	No	
Mr Neil Thomas (Tom) O'Kane	Aviation Safety Consultant	No	
Dr Joanne Philpot	Consultant Paediatrician	Yes	
Dr Mike Proven (Vice Chair)	Coordinator for QA in Research	Yes	
Ms Ann Quinn (Alternate Vice Chair)	Social Worker	No	
Dr Deborah Scholey	Regulatory Affairs Consultant	Yes	
Mr Donald Scott-Collett	Lead Pharmacist for Elderly Care, Neuro-rehabilitation, Dermatology and Clinical Governance	Yes	
Dr John Andrew Sutton	Medical Director	Yes	
Ms Susan Tonks	Senior Research Support Associate	Yes	
Mrs Helen Turner	Clinical Study Manager	Yes	

Also in Attendance:

Name	*Position (or reason for attending)*
Miss Charlotte Ferris	Approvals Administrator
Ms Nabeela Iqbal	Approvals Specialist
